# Obstructive sleep apnea hypopnea syndrome: Protocol for the development of a core outcome set

**DOI:** 10.1097/MD.0000000000021591

**Published:** 2020-08-21

**Authors:** Pingping Wanyan, Jianqin Wang, Wenge Wang, Yuke Kong, Yaojun Liang, Wei Liu, Qin Yu

**Affiliations:** a the First Clinical Medical College of Lanzhou University; b the Second Hospital of Lanzhou University; c the First Hospital of Lanzhou University, Lanzhou, China.

**Keywords:** clinical trials, consensus methods, core outcome set, Delphi survey, domains, obstructive sleep apnea-hypopnea syndrome

## Abstract

**Background:**

Obstructive sleep apnea hypopnea syndrome (OSAHS) is a common, chronic sleep disease. As the incidence of OSAHS increases, it has seriously threatened people's health. There have been an increasing number of clinical trials of OSAHS in recent years. However, the clinical trials of OSAHS have heterogeneous outcomes, surrogate outcomes, subjective outcomes, and composite outcomes, as well as the lack of endpoints or patient perspectives. The best method is to develop a core outcomes sets (COSs) for OSAHS's clinical trials.

**Methods:**

The development of COSs of OSAHS will include 5 stages:

(1) set up a Steering Group, defining the scope of this COSs;
(2) identifying potential core outcomes by systematic review and semi-structured interviews;
(3) identifying final COSs by consensus process, includes Delphi survey and face-to-face meeting;
(4) identification and evaluation of instruments used to measure the final COSs;
(5) promotion and updates.

**Results:**

The results of our study will be published in a peer-reviewed journal.

**Discussion:**

The development of the COSs of OSAHS will improve the design and operation of OSAHS clinical trials to conform to international standards and ensure the credibility of the outcomes. In addition, this study will involve different stakeholder groups to help ensure that the developed COSs will be suitable and well accepted.

**Trial registration number:**

1544.

## Abstract 2

Supplemental Digital Content is available in the text

## 1 Introduction

Obstructive Sleep Apnea-Hypopnea Syndrome (OSAHS) is a common, chronic sleep disease, which a person frequently apnea and hypoventilation during his or her sleep.^[1]^ This is due to the lack of exercise tension of the tongue and/or airway dilators during sleep, resulting in obstruction of the upper respiratory tract. Its clinical characteristics include snoring (irregular snoring), patients consciously hold their breath (even being repeatedly awakened), often accompanied by a series of symptoms such as increased nocturnal, morning headache, dizziness, or pharyngeal dryness.^[2]^ And clinical evidence also suggests that OSAHS may contribute to the development of systemic hypertension, cardiovascular disease, and abnormalities in glucose metabolism.^[3–5]^ Moreover, OSAHS is insidious, and patients are often unaware of its developments, which seriously threaten people's health. The prevalence of OSAHS male is estimated to be 3% to 7%, and for female 2% to 5% in USA.^[6]^ There is currently a lack of epidemiological statistics on the disease in China, and the prevalence is estimated to be higher than 4%. Obesity is considered as the main reason for the occurrence of OSAHS.^[7]^ With the increase in overweight and obese people, the incidence of the disease will continue to rise.

Early recognition and appropriate therapy are the primary options for managing the disease.^[8]^ The overnight polysomnogram is the standard diagnostic test for OSAHS.^[6]^ It can simultaneously record multiple physiological signals during sleep, such as electroencephalogram, electrooculogram, electromyogram, oronasal airflow, and oxyhemoglobin saturation. As a chronic disease, the treatment of OSAHS is mainly to relieve upper airway obstruction, and needs to education and follow up patients continuously, and adjust the treatment strategy in time to ensure the efficacy.^[9]^ Lifestyle modifications, such as weight loss, alcohol sedative-avoidance, smoking cessation, avoidance of sleep deprivation, will decrease both the symptoms of OSAHS and the comorbid conditions.^[7,10]^ In addition, Chinese medicine has also become an effective treatment for OSAHS.^[11,12]^ All in all, there have been an increasing number of clinical trials of OSAHS in recent years. However, like clinical trials in other fields,^[13–15]^ the clinical trials of OSAHS also have heterogeneous outcomes, surrogate outcomes, subjective outcomes, and composite outcomes, as well as lack of endpoints or patient perspectives.

The best method to improve the reporting of outcomes is to develop a core outcomes sets (COSs), that is, the minimum set of outcomes in which all studies in a particular situation are scientifically agreed and reported.^[16]^ The COSs must be measurable and relevant. Involving patients and other key stakeholders are the key to achieving relevance. Studies have shown that the COS can make the reporting of outcomes of the study more standardized.^[17,18]^ With the Core Outcome Measures in Effectiveness Trials (COMET) Initiative founded and committed to the development, implementation, dissemination and update of COSs by published the consensus-based standards for the selection of health measurement instruments (COSMIN), the development and implemented of COSs has become an inevitable trend.^[19]^

To date, there is currently no available COSs for OSAHS's clinical trials. The first aim of this study is to develop a COS for OSAHS's clinical trials. The second aim is to select a measurement instrument for each outcome included in the COSs with the method recommended by the COMET initiative after completing the COSs. Finally, we will focus on the promotion of this COSs.

## 2 Methods

**Figure 1:**
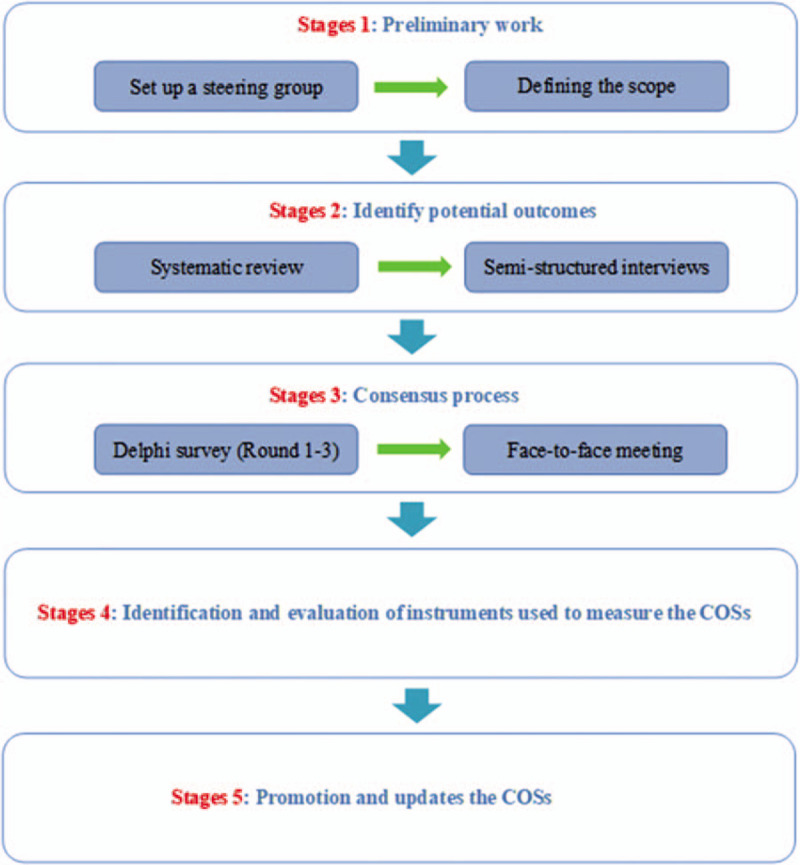
Flowchart of the study design.

The development of this COS follows COMET and COS-standard for Development core outcomes sets -standard for developmerecommendations.^[19,20]^ At the same time make appropriate adjustments according to the actual situation. After completing the registration, there are 5 stages:

(1) set up a Steering Group, defining the scope of this COSs;
(2) identifying potential core outcomes by systematic review and semi-structured interviews;
(3) identifying final COSs by consensus process, includes Delphi survey and face-to-face meeting;
(4) identification and evaluation of instruments used to measure the final COSs;
(5) promotion and updates. See detail in Figure 1.

### 2.1 Prospective registrations

This study has been registered on the COMET database (Number: 1544). (http://www.comet-initiative.org/Studies/Details/1544)

### 2.2 Steering Group

We plan to form a national Steering Group to support the development of this COSs. The Steering Group includes 3 expertis in the fields of OSAHS and 1 expert in COSs development. In addition, this study also included 3 fellows and identifies a leader among them, in line with core outcomes sets -standard for developme recommendations.^[20]^ Members of the Steering Group and fellows were contacted by email and telephone regarding key decisions.

### 2.3 Scope of the core outcome set

The aim of this article is to present a protocol for a study to develop a COSs for clinical trials in the patients (≥18 years) with OSAHS. Gender and race of patients are not limited. We foresee that the COSs will be used in OSAHS clinical trials in various interventions.

### 2.4 Identifying potential core outcomes

The potential core outcomes will be generated by extracting outcomes from the published literature on the OSAHS through a systematic review. In addition, a series of semi-structured interviews with individuals in OSAHS can complement the potential core outcomes.

#### 2.4.1 Systematic review

##### 2.4.1.1 Research question

What outcomes are reported in the medical literature for OSAHS?

##### 2.4.1.2 Inclusion criteria and exclusion criteria

The inclusion and exclusion criteria for the literature are shown in Table 1.

**Table 1:**
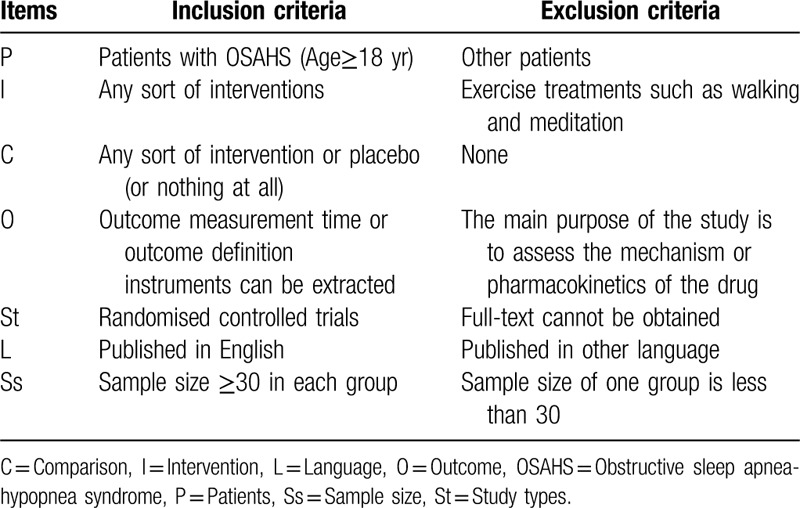
The inclusion and exclusion criteria of systematic review.

##### 2.4.1.3 Search strategy

We plan to search the following databases: PubMed, Embase, Cochrane library, and Web of Science. A comprehensive search strategy will be used (See detail in Supplemental Digital Content (Appendix Table 1), Key terms used to guide the search will include “obstructive sleep apnea”, “upper airway resistance sleep apnea syndrome”, “OSAHS” and “randomized controlled trials”. Language restrictions will not be applied to the search strategy; however, selection of relevant articles will be restricted to English language publications.

##### 2.4.1.4 Study selection

The initial selection of studies identified in the search will be conducted using predetermined review inclusion/exclusion criteria. Firstly, 2 reviewers will independently assess the titles and abstracts of selecting studies. Secondly, full texts of studies which meeting the inclusion criteria or uncertainty regarding inclusion of title/abstract screening will be retrieved and reviewed with final decisions by the same reviewers. The third independent reviewer will be consulted to resolve the disagreement.

##### 2.4.1.5 Data extraction

Data will be extracted independently by 2 review authors and checked by a third author. Any unclear and/or unavailable data will be identified by contacting study authors. The following data will be extracted from each included study: author, year and journal of publication, intervention(s), each effectiveness outcome reported, whether the outcome was defined or not, the definition used, the indicators and/or tool(s) used to operationalism or measure the outcome, the time point or period of outcome measurement, and how the outcome was reported.

##### 2.4.1.6 Data analysis and presentation

Two reviewers will independently standardize the outcomes and categorize them into different domains. The following process will be followed:

(1) adjust the outcomes to standard terminology (If no relevant terminology, the Steering Group will be consulted.);
(2) composite outcomes will be divided into a single outcome;
(3) define duplicate outcomes will be deleted;
(4) categorize outcomes into different domains (such as mortality, quality of life, treatment satisfaction and adverse event);
(5) the frequency of each outcome and the number of outcomes in each domains will be counted;
(6) reviewers will check and resolve the disagreement through discussion with the other reviewer;
(7) the Steering Group will conduct a final review.

#### 2.4.2 Semi-structured interviews

Adult patients with OSAHS will be interviewed to obtain outcomes that the patient considers important. This study plans to interview 10 patients (men and women half), they must meet the following conditions:

(1) no communication disorders or mental illness;
(2) medical history more than 3 years;
(3) agree to be interviewed. By comparing the outcomes of the systematic review, the 2 reviewers will determine which outcomes are new. Whether it will be included in the potential core outcomes should be decided by the Steering Group.

### 2.5 Determining core outcomes

Following the guidance of COMET, like other similar studies, this study will determine the final core outcomes through Delphi survey and face-to-face meeting.

#### 2.5.1 Delphi survey

We will use a Delphi survey to develop consensus on the core outcomes from those identified in the systematic reviews and semi-structured interviews. The advantage of this technique over a roundtable meeting is that it avoids situations where some people can lead the discussion or situations where other individuals feel obligated to agree with the opinions of more senior members. In order to improve efficiency, the questionnaire will be completed online using appropriate online survey design software.^[21]^ A minimum of 3 Delphi rounds is planned. There is currently no standard sample size calculation method in the Delphi process, but our goal is a total of 180 participants, including at least 30 patients (Confirmed cases from tertiary hospitals aged over 18 with a history of more than 3 years.).^[22]^ We will strive to increase the response rate of the participants.

The participants are all stakeholders. In addition to patients, we plan to include the following 3 groups of people:

(1) clinicians: respiratory physician, cardiologist, otolaryngologist, general practitioner, doctor of traditional Chinese medicine, nurse;
(2) researchers: experts in respiratory diseases at domestic and foreign medical schools, respiratory specialists in various research institutions;
(3) policy-makers: national, provincial, municipal, county, and other levels of health management personnel, some health policy-makers outside of China will also be invited. Potential participants will determine whether they are willing to participate in the Delphi survey via email or phone.

A preliminary list of potential outcomes will be produced by reviewers, and will be reviewed by the Steering Group. Participants are asked to evaluate the importance of the listed items. Participants can also choose to add other items they think are missing and score each item added. The detailed process will proceed as following.

Round 1

In Delphi survey round 1, the first is to obtain the participant's name, email address and their location in order to provide every participant with a unique identifier. Secondly, participants will be asked to identify the stakeholder group. The questionnaires based on the preliminary list of potential outcomes will contain lay terminology alongside clinical terms to assist participants (especially patients) in understanding complex terminology. In the survey, the order of outcomes and the order of domains within outcomes will be randomized. The participants will be asked to rate the potential outcomes on a 9-point Likert-scale.^[23]^ Scores will be grouped into 1 to 3 (signifies an outcome is of limited importance), 4 to 6 (important but not critical), and 7 to 9 (critical). If a participant feels that they did not understand a certain outcome, they will be able to select unable to score. In addition, each participant is allowed to add 2 additional outcomes and add a score to each added outcome. The Delphi survey round 1 will be required to complete the questionnaire within 3 weeks. A reminder email will be sent on the second weekend. Non-responders will not be invited to participate in round 2.

Two reviewers will independently analyze and collate the responses from the first round. Any disagreements will be resolved through discussion or determined by the Steering Group. The responses of round 1 will be summarized using descriptive statistics, including the additional outcomes. For each outcome, the number of participants who have scored the outcome and the distribution of scores will be summarized by reviewers. All outcomes will be carried forward to round 2. In addition, participants in the round 2 can also see the distribution of score of each outcome.

Round 2

The purpose of the round 2 will be enabled participants to reflect on their score based on the views of stakeholders on peers and score the outcomes again. As in round 1, participants will be asked to rate all outcomes again on the 9-point Likert-scale. This round 2 will also be required for completion within 3 weeks. Participants will be reminded by email on the weekend of the second week.

If an outcome scored “critical” by more than 70% of participants from any stakeholder group and “limited importance” by less than 15% of participants from any stakeholder group (See detail in Table 2), it will be carried forward for further consideration in round 3. Like the previous study, the theoretical basis for this study to do so is that for an outcome to be included in COS, it requires agreement by the majority regarding the critical importance of the outcome, with only a small minority considering it to have little importance.^[24]^ The purpose of this round of the Delphi survey is to determine the most important outcomes, and all other outcomes will be discarded. As in the previous round, 2 reviewers will independently conduct statistics and analysis. Any disagreements will be resolved through discussion or determined by the Steering Group.

**Table 2:**
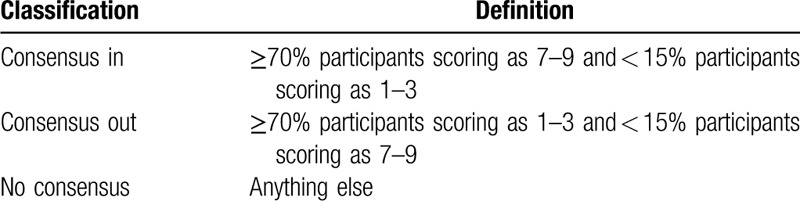
Definitions of consensus.

Round 3

In round 3, only participants who have responded to at least 40% of the outcomes in the round 2 will be eligible to continue the survey. Participants will see the outcomes included by the round 2 survey, including the number of participants scored and score distribution for each outcomes. The 9-point Likert-scale will be used again. And this round survey also requires completion within 3 weeks.

If an outcome still scored “critical” by more than 70% of participants from any stakeholder group and “limited importance” by less than 15% of participants from any stakeholder group (See detail in Table 2), it will be carried forward for face-to-face meeting. All statistical analysis processes will require the same as the round 2.

#### 2.5.2 Face-to-face meeting

The purpose of this face-to-face meeting is to discuss the divergent outcomes in the round 3 of Delphi survey, and to verify and agree on the list of final outcomes that constitute COS. If they still disagreements on some outcomes, they will resolve it by voting. Similar to the Delphi stage, outcomes will be considered as “consensus in” if 70% of the consensus meeting participants votes in favors of the outcomes to be included in the COS. The planned meeting time is half a day. In order to achieve effective consensus, the participants consist of 2 parts: 1 facilitator and 13 stakeholders. The facilitator will not be able to participate in the discussion. Firstly, these stakeholders whom must complete all rounds of the Delphi study. The 13 stakeholders in this study will be distributed as follows: 4 patients, 4 clinicians, 4 researchers, and 1 policy-makers. All participants will be paid a certain amount, such as travel expenses and accommodation fee.

### 2.6 Selection of instruments for each final COSs

Following completion of the identification of final COSs, 2 reviewers will systematically review the instruments for each final COSs in clinical trials for OSAHS. If the outcome only has 1 instrument, then this instrument is the final instrument. If there are multiple instruments for a outcomes, the identified outcome instruments will be evaluated according to the 9 measurement properties identified by the COSMIN initiative:^[19]^ Internal consistency, reliability, measurement error, content validity, structural validity, hypotheses testing, cross-cultural validity, criterion validity, and responsiveness. The instrument with the highest score will be selected.

### 2.7 Dissemination strategies

The final COSs will be promoting primarily via publications, various conferences, professional societies, and our institutions social media platforms. Such as the publications, the first step will be to publish the protocol for the development of the COS. Secondly, a paper that detailing the COSs to be used for each domain in OSAHS trials will be published. Finally, the recommended COS will be published in the COMET database.

### 2.8 Update

As we all know, as medical research continues, understanding of disease, diagnosis, treatment, and evaluation will be updated. Therefore, the COSs of OSAHS also needs constant evaluation and upgrading in accordance with the latest achievements of clinical research. In the promotion process, it is necessary to pay attention to the shortcomings of the COSs of OSAHS at any time. It should then be revised in accordance with standard procedures based on the number and importance of these shortcomings. Only by continuously adding new outcomes and new instruments can we ensure the practicality and advancement of the COS of OSAHS.

## 3 Discussions

Development of a COS is an iterative approach. Firstly, determine which outcomes that should be measured and reported. Secondly, determine to which outcome measurement instruments should be used to measure the COSs. To date, there has been no COS for OSAHS clinical trials. The development of COSs will improve the design and operation of OSAHS clinical trials to conform to international standards and ensure the credibility of the outcomes. In addition, this study will involve different stakeholder groups to ensure that the developed COS will be suitable and well accepted. In short, this study will ensure the consistency of important outcomes, reduce reporting bias, improve the comparability of future studies, improve the methodological quality of OSAHS clinical trials, and the practicality of study outcomes. At the same time, medical resources can also be saved.

## 4 Ethical approval

Ethics approval has been granted by the First Hospital of Lanzhou University Human Ethics Committee (LDYYLL2020-225). Participation into the Delphi surveys is optional and each participant will be required to give informed consent.

## 5 Trial status

The study protocol is version 1.0 (May 1, 2020). Recruitment commenced on May 4, 2020 and is expected to be complete before June 4, 2020. The study is expected to take 3 months once recruitment is complete.

## Acknowledgments

We thank Ming Liu (Evidence Based Medicine Center, School of Basic Medical Sciences, Lanzhou University) for helping us complete the registration of the study in the COMET database.

## Author Contributions

PP Wanyan composed working documents for the process in the initial phase. PP Wanyan has made the first draft of all sections of the protocol mainly under supervision from Q Yu. PP Wanyan, JQ Wang, WG Wang, YK Kong, YJ Liang, W Liu and Q Yu participated in several telephone conferences where the method has been discussed. PP Wanyan conducted the systematic reviews that formed the basis of the candidate items. JQ Wang, W Liu and WG Wang were responsible for the qualitative interviews in China. YK Kong, YJ Liang and Q Yu conducted the qualitative interviews outside of China. PP Wanyan wrote the first draft of the manuscript, and all authors provided comments and approved the final version.

**Data curation:** Ping-Ping Wanyan and Qin Yu.

**Formal analysis:** Jian-Qin Wang, Wen-Ge Wang.

**Funding acquisition:** Qin Yu.

**Methodology:** Ping-Ping Wanyan and Yu-Ke Kong.

**Resources:** Yao-Jun Liang, Ping-Ping Wanyan and Wei Liu.

**Sofeware:** Ping-Ping Wanyan and Jian-Qin Wang.

**Writing-original draft:** Ping-Ping Wanyan.

**Writing-review and editing:** Ping-Ping Wanyan and Qin Yu.

## Supplementary Material

Supplemental Digital Content
