# Supplementary material for: Obstructive sleep apnea hypopnea syndrome: Protocol for the development of a core outcome set
Source: Medicine (Baltimore). 2020 Aug 21;99(34):e21591. doi: 10.1097/MD.0000000000021591 (PMC7447502; doi:10.1097/MD.0000000000021591)
Supplement: Supplemental Digital Content [file medi-99-e21591-s001.docx]

**Search strategy**

**PubMed**

#1 "Sleep Apnea, Obstructive"[Mesh]

#2 "obstructive sleep apnea"[title/abstract] OR "obstructive sleep apnoea"[title/abstract] OR "obstructive sleep apnoea hypopnoea syndrome"[title/abstract] OR

"obstructive sleep apnea hypopnea syndrome"[title/abstract] OR "obstructive sleep apnea syndrome"[title/abstract] OR OSAHS[title/abstract] OR "sleep apnea hypopnea syndrome"[title/abstract] OR "upper airway resistance sleep apnea syndrome"[title/abstract]

#3 #1 OR #2

#4"Randomized Controlled Trials as Topic"[Mesh] OR "Randomized Controlled Trial" [Publication Type]

#5 "Randomized Controlled Trial"[title/abstract] OR "Randomized Controlled Trials"[title/abstract] OR "Controlled Clinical Trials, Randomized"[title/abstract]

#6 #4 OR #5

#7 #3 AND #6

**Embase**

#1 "Obstructive Sleep Apnea"/exp

#2 "obstructive sleep apnea":ab,ti OR "obstructive sleep apnoea":ab,ti OR "obstructive sleep apnoea hypopnoea syndrome":ab,ti OR

"obstructive sleep apnea hypopnea syndrome":ab,ti OR "obstructive sleep apnea syndrome":ab,ti OR OSAHS:ab,ti OR "sleep apnea hypopnea syndrome":ab,ti OR "upper airway resistance sleep apnea syndrome":ab,ti

#3 #1 OR #2

#4 "Randomized Controlled Trial"/exp

#5 "Randomized Controlled Trial":ab,ti OR "Randomized Controlled Trials":ab,ti OR "Controlled Clinical Trials, Randomized":ab,ti

#6 #4 OR #5

#7 #3 AND #6

**Cochrane Library**

#1 MeSH descriptor: [Sleep Apnea, Obstructive] explode all trees

#2 "obstructive sleep apnea":ab,ti,kw OR "obstructive sleep apnoea":ab,ti,kw OR "obstructive sleep apnoea hypopnoea syndrome":ab,ti,kw OR

"obstructive sleep apnea hypopnea syndrome":ab,ti,kw OR "obstructive sleep apnea syndrome":ab,ti,kw OR OSAHS:ab,ti,kw OR "sleep apnea hypopnea syndrome":ab,ti,kw OR "upper airway resistance sleep apnea syndrome":ab,ti,kw

#3 #1 OR #2

#4 MeSH descriptor: [Randomized Controlled Trial] explode all trees

#5 "Randomized Controlled Trial":ab,ti,kw OR "Randomized Controlled Trials":ab,ti,kw OR "Controlled Clinical Trials, Randomized":ab,ti,kw

#6 #4 OR #5

#7 #3 AND #6

**Web of science**

(TS="obstructive sleep apnea" OR "obstructive sleep apnoea" OR "obstructive sleep apnoea hypopnoea syndrome" OR "obstructive sleep apnea hypopnea syndrome" OR "obstructive sleep apnea syndrome" OR OSAHS OR "sleep apnea hypopnea syndrome" OR "upper airway resistance sleep apnea syndrome") AND TS=("Randomized Controlled Trial" OR "Randomized Controlled Trials" OR "Controlled Clinical Trials, Randomized")
